# Spatiotemporal Remodeling of Neural Excitability Following Ischemic Stroke: Regional Mechanisms From the Infarct Core to Brainstem–Spinal Output Systems: A Structured Narrative Review

**DOI:** 10.1155/np/3095088

**Published:** 2026-10-29

**Authors:** Lu Li, Hongmei Shi, Shanshan Zeng, Lin Huang, Miaomiao Yang, Xiongxing Sun, Dahua Wu, Le Xie

**Affiliations:** ^1^ Department of Neurology Hunan Provincial Hospital of Integrated Traditional Chinese and Western Medicine (The Affiliated Hospital of Hunan Academy Traditional Chinese Medicine) Changsha 410006 Hunan China; ^2^ Graduate School Hunan University of Chinese Medicine Changsa 410208 Hunan China hnctcm.edu.cn

**Keywords:** ischemic stroke, neural excitability, neural plasticity, peri-infarct cortex, reticulospinal tract, spreading depolarization

## Abstract

Changes in neural excitability after ischemic stroke affect lesion growth, recovery of function, and long‐term motor problems. People often think of these changes as just an increase or decrease in neural activity. This view does not explain why excitability changes are different at different stages, brain regions, or mechanisms. It also does not explain why these changes can cause injury, help recovery, or lead to maladaptation. In the acute phase, the main areas involved are the infarct core and the ischemic penumbra. Here, excitability problems include membrane instability, prolonged depolarization, spreading depolarizations (SDs), changes in the fluid environment, and abnormal neurovascular responses. These problems together create harmful excitability that makes the lesion bigger. In the subacute phase, the dominant substrates shift toward the peri‐infarct cortex, ipsilesional primary motor cortex, and bilateral sensorimotor networks. Here, excitability changes are defined by excitatory–inhibitory rebalancing, reduction of excessive tonic inhibition, restoration of cortical responsiveness, and experience‐dependent network reorganization, forming restorative excitability that supports neural plasticity and functional recovery. During the subacute phase, excitatory–inhibitory rebalancing becomes a key synaptic mechanism of recovery, involving glutamatergic transmission, γ‐aminobutyric acid (GABA)ergic tonic and phasic inhibition, synaptic scaling, dendritic remodeling, and activity‐dependent refinement of surviving peri‐infarct circuits. In the chronic phase, in a subset of patients, the dominant substrates further shift toward the brainstem‐spinal output system, where compensatory recruitment of alternative descending pathways may contribute to abnormal synergies, spasticity, and low‐resolution motor control, representing maladaptive hyperexcitability. We therefore argue that poststroke excitability should not be interpreted within a static high‐/low‐excitability dichotomy but defined by when it occurs, where it is expressed, and at which mechanistic level it predominates.

## 1. Introduction

Stroke disrupts the excitation–inhibition balance across multiple levels of the nervous system. However, poststroke excitability is neither uniformly increased nor intrinsically harmful; its mechanisms and functional consequences vary with time, anatomical location, and neural substrate [1–5]. During acute ischemia, energy failure and ionic‐gradient collapse cause sustained cellular depolarization, glutamate excitotoxicity, and cytotoxic swelling [6–14]. Recurrent spreading depolarizations (SDs) can further increase metabolic demand and promote infarct expansion, particularly when neurovascular responses are insufficient or paradoxically constrictive [15–42]. In this early phase, excessive excitability therefore primarily represents an injury‐amplifying process.

As stroke evolves from injury to repair, excitability acquires a different functional role. During the subacute phase, changes in GABAergic inhibition, activity‐dependent trophic signaling, axonal sprouting, dendritic remodeling, and cortical map reorganization create a transient environment permissive for plasticity [4, 43–61]. Reduced excessive tonic inhibition may facilitate peri‐infarct responsiveness and motor recovery, whereas appropriately regulated phasic inhibition may stabilize reorganizing circuits [43, 46, 55, 62, 63]. Poststroke recovery should therefore not be understood as generalized excitation or disinhibition but as a temporally constrained rebalancing of neural activity.

At the systems level, motor outcome depends on the interaction between cortical excitability, network connectivity, and the integrity of descending pathways. The recovery of ipsilesional motor activity is generally associated with better outcomes when the corticospinal tract (CST) remains sufficiently preserved [64–73]. By contrast, severe corticospinal injury may increase reliance on contralesional corticoreticulospinal and other indirect descending pathways. Although these pathways can support gross force and proximal movement, their excessive recruitment may contribute to abnormal synergies, impaired motor fractionation, and spasticity [74–91]. Thus, behavioral improvement may reflect either restitution through residual corticospinal pathways or compensation through less selective brainstem motor systems.

In this review, we integrate clinical, in vivo preclinical, and mechanistic evidence to propose a phase‐dependent framework of poststroke excitability comprising acute injurious depolarization, subacute restorative excitability, and divergent chronic motor output. Rather than asking whether excitability should be globally suppressed or enhanced, the clinically relevant questions are which neural element is excitable, where and when the change occurs, and whether its output supports restitution or compensation. Distinguishing membrane, synaptic, cortical, network, descending pathway, and spinal excitability may reconcile apparently conflicting findings and provide a basis for phase‐specific biomarkers, neuromodulation, and rehabilitation strategies.

## 2. Review Methodology

### 2.1. Review Design

This article is a narrative review informed by a structured and reproducible literature search. It was designed to provide a mechanistically organized synthesis of clinical and preclinical evidence concerning the spatiotemporal remodeling of neural excitability after ischemic stroke. Because the aim was conceptual integration across heterogeneous electrophysiological, molecular, imaging, histological, and behavioral evidence rather than formal effect estimation or comprehensive evidence mapping, the article was not conducted as a systematic review or scoping review and does not claim compliance with PRISMA 2020 or PRISMA‐ScR.

### 2.2. Information Sources and Search Strategy

PubMed/MEDLINE and Web of Science Core Collection were searched from database inception to May 14, 2026. The final search was performed on July 25, 2026. The electronic search was restricted to publications in English. No restriction on publication year was applied. A modular search strategy was used because the review addressed distinct but interconnected mechanisms across the temporal course of ischemic stroke. Terms for ischemic stroke were combined with three principal mechanistic domains: (1) acute injurious excitability, including membrane depolarization, ionic‐gradient failure, SD, ischemic penumbra, cellular swelling, and altered neurovascular coupling; (2) subacute restorative excitability, including peri‐infarct plasticity, glutamatergic and GABAergic signaling, tonic and phasic inhibition, cortical reactivity, sensorimotor‐network reorganization, corticospinal‐tract integrity, and experience‐dependent plasticity; and (3) chronic maladaptive excitability, including corticoreticulospinal and reticulospinal recruitment, spinal‐circuit excitability, stretch‐reflex hyperexcitability, abnormal motor synergies, co‐contraction, and poststroke spasticity.

Controlled‐vocabulary terms and free‐text terms were combined as appropriate for each database. Medical Subject Headings were used in PubMed/MEDLINE, topic fields, and proximity operators in Web of Science Core Collection. Synonymous terms within each concept were combined using “OR,” whereas the ischemic‐stroke and mechanistic domains were combined using “AND.” Phrase searching, truncation, spelling variants, and proximity operators were used where appropriate.

The complete database‐specific search strategies and the numbers of records retrieved are provided in Figure S1. Literature identification and study‐selection process. In addition, the reference lists of eligible studies and relevant reviews were manually examined, and forward citation searching was performed for key mechanistic studies.

### 2.3. Eligibility Criteria

Publications were eligible for the core evidence synthesis if they met the following criteria: (1) They investigated adult ischemic stroke or an established in vivo model of focal cerebral ischemia; (2) they examined membrane, synaptic, network, cortical, descending‐pathway, or spinal‐circuit excitability; (3) they addressed acute tissue injury, poststroke neural plasticity, motor‐system reorganization, functional recovery, abnormal motor synergies, or spasticity; and (4) they reported relevant electrophysiological, neurophysiological, imaging, histological, molecular, behavioral, neurological, or motor outcomes.

Eligible clinical evidence included studies of cortical or subcortical ischemic infarction and anterior‐ or posterior‐circulation ischemic stroke. Mixed‐stroke cohorts were included only when ischemic‐stroke findings were separately extractable or when a clearly defined ischemic‐stroke subgroup was reported. Eligible preclinical models included transient or permanent focal cerebral ischemia, middle cerebral artery occlusion, photothrombotic stroke, and focal cortical ischemia.

Studies were excluded from the core evidence synthesis if they (1) focused exclusively on intracerebral hemorrhage, subarachnoid hemorrhage, transient ischemic attack without cerebral infarction, global cerebral ischemia, neonatal hypoxic–ischemic injury, traumatic brain injury, spinal cord injury, or another nonstroke neurological condition; (2) investigated only acute recanalization, thrombolysis, thrombectomy, or neuroprotection without evaluating neural excitability, plasticity, or functional recovery; (3) did not report ischemic‐stroke data separately from other stroke subtypes; (4) were conference abstracts, protocols, editorials, letters, or isolated case reports; or (5) did not provide information relevant to the predefined spatiotemporal or mechanistic domains of the review.

Foundational nonstroke neurophysiological and in vitro studies were cited only when required to explain basic receptor function, membrane physiology, or the organization of descending pathways. These publications were classified as contextual mechanistic evidence and were not interpreted as direct evidence of poststroke excitability or recovery.

### 2.4. Study Selection and Evidence Classification

Search results were imported into EndNote 21, and duplicate records were removed. Two reviewers (Shanshan Zeng and Miaomiao Yang) independently screened titles and abstracts. Potentially eligible publications were subsequently evaluated in full text. Disagreements were resolved through discussion and, when required, consultation with a third author (Hongmei Shi). Reasons for exclusion at the full‐text stage were recorded.

Eligible publications were classified according to (1) evidence type—human clinical, in vivo preclinical, or contextual mechanistic evidence; (2) disease phase—acute, subacute, or chronic; (3) anatomical level—infarct core/penumbra, peri‐infarct cortex, distributed sensorimotor network, descending motor pathway, or spinal output system; and (4) mechanistic level—membrane, synaptic, network, or descending motor‐drive excitability.

Because the purpose of this narrative review was conceptual and mechanistic integration rather than pooled effect estimation, the evidence was synthesized qualitatively.

The database searches identified 7081 records, and 59 additional records were identified through backward and forward citation searching. After the removal of 1412 duplicates, 5728 records underwent title and abstract screening. Of these, 573 publications were assessed in full text, and 5155 were excluded for the eligibility criteria. A total of 118 publications were included in the final synthesis, comprising 28 human clinical studies, 28 in vivo preclinical studies, and 62 contextual mechanistic publications.

## 3. A Multilevel Definition and General Framework of Excitability Following Ischemic Stroke

### 3.1. Poststroke Excitability Is a Multilevel Concept Rather Than a Single Electrophysiological Measure

Changes in excitability following ischemic stroke are often invoked to describe a broad spectrum of neurophysiological phenomena spanning acute tissue injury, functional recovery, and chronic motor complications [1, 2]. Early conceptual work by Carmichael highlighted the dynamic and bidirectional nature of poststroke excitability, and this “yin and yang” view of stroke progression represents a direct antecedent of the present tripartite framework [3]. However, without a hierarchical definition, excitability is easily reduced to a simple increase or decrease in neural activity [1]. This simplification obscures the fact that poststroke outcomes do not depend on a unidirectional change in activity within a single region but on how the nervous system redistributes its capacity to respond to, amplify, propagate, and transform inputs into functional outputs within a specific metabolic and structural context [2–5]. In this review, excitability is therefore not used in the narrow sense of firing intensity alone but as a multilevel property encompassing neurons, local circuits, distributed networks, and descending output systems [1, 2].

### 3.2. The Four Levels of Excitability Mechanisms

Poststroke excitability should be understood as a multilevel phenomenon rather than as a single electrophysiological variable. At the membrane level, excitability reflects ion gradients, resting membrane potential, channel function, and depolarization threshold. At the synaptic level, it reflects the balance between glutamatergic excitation and GABAergic inhibition, synaptic gain control, dendritic integration, and activity‐dependent plasticity. At the circuit level, it reflects the recruitment and reweighting of local and descending pathways, including corticospinal, corticoreticular, reticulospinal, and spinal reflex circuits. At the network level, it reflects interregional connectivity, interhemispheric interactions, and distributed sensorimotor integration. These levels are interdependent but not interchangeable, and each may contribute differently across the acute, subacute, and chronic stages after stroke.

#### 3.2.1. Membrane Excitability

The membrane excitability represents the most fundamental level of poststroke excitability change [6, 7]. It refers to the ability of cells to maintain transmembrane ionic gradients, resting membrane potential, and depolarization–repolarization responses to stimulation [8]. This level depends not only on ion channel function but also on adenosine triphosphate (ATP) supply, Na^+^/K^+^–ATPase activity [9], and ion–water transport processes involved in cell volume regulation [10]. In the infarct core and severely hypoperfused tissue, excitability abnormalities at this level do not indicate functional hyperexcitability but rather electrochemical homeostasis failure, characterized by ionic gradient collapse, sustained depolarization, electrical silence, and early cellular swelling [4, 9, 11].

#### 3.2.2. Synaptic Excitability and Excitatory–Inhibitory Rebalancing

At the synaptic level, excitability refers to the dynamic balance between excitation and inhibition within local circuits and the responsiveness of these circuits to incoming input [1, 12]. Following ischemic stroke, glutamatergic excitation and GABAergic inhibition do not change synchronously or uniformly but are remodeled in a stage‐, region‐, and cell type–dependent manner [4]. During the acute phase, synaptic abnormalities are dominated by excitotoxicity, with excessive glutamate release, impaired uptake, and receptor overactivation contributing to tissue injury [13, 14]. In the subacute phase, the focus shifts from excitotoxic injury to synaptic remodeling and excitatory–inhibitory rebalancing. In peri‐infarct circuits, excessive extrasynaptic GABA_A receptor–mediated tonic inhibition may suppress cortical map reorganization and limit functional recovery [43]. However, GABAergic inhibition is not uniformly detrimental. Phasic inhibition, mediated mainly by synaptic GABA_A receptors, shapes spike timing and firing precision, whereas tonic inhibition, mediated largely by extrasynaptic GABA_A receptors, regulates baseline neuronal gain and plasticity thresholds [12]. Moreover, enhanced phasic GABAergic signaling during the repair phase may support, rather than suppress, recovery [46]. At the same time, selective excitatory and structural plasticity also contribute to restorative reorganization. A‐amino‐3‐hydroxy‐5‐methyl‐4‐isoxazolepropionic acid receptor (AMPAR)–brain‐derived neurotrophic factor (BDNF) signaling, cAMP response element–binding protein (CREB)–related pathways, dendritic spine remodeling, synaptic strengthening, and activity‐dependent remapping may support the rewiring of spared cortical and corticospinal circuits [44, 45, 60, 92] (Figure 1). In other words, the disinhibition associated with recovery is more akin to a controlled, plasticity‐permissive reorganization of neural circuits than to a pathological increase in neuronal firing [4, 43, 46].

**Figure 1:**
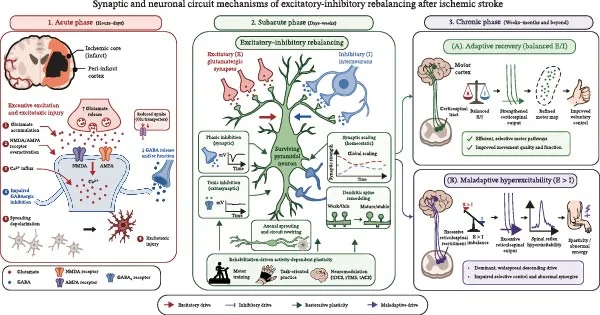
Synaptic and neuronal circuit mechanisms of excitatory–inhibitory rebalancing after ischemic stroke. This figure shows stage‐dependent changes in synaptic and circuit excitability after ischemic stroke. In the acute phase, excessive glutamate accumulation, NMDA and AMPA receptor overactivation, Ca^2+^ influx, impaired GABAergic inhibition, and spreading depolarization contribute to excitotoxic injury. In the subacute phase, peri‐infarct circuits undergo excitatory–inhibitory rebalancing. Controlled reduction of excessive tonic inhibition, preservation of phasic inhibition, synaptic scaling, dendritic spine remodeling, axonal sprouting, and rehabilitation‐driven plasticity support circuit rewiring. In the chronic phase, balanced excitatory–inhibitory regulation and strengthened corticospinal output promote adaptive motor recovery. By contrast, relative dominance of reticulospinal output and spinal reflex hyperexcitability contributes to abnormal synergies and spasticity [12, 43–46]. Red indicates excitatory drive, blue indicates inhibitory drive, green indicates restorative plasticity, and purple indicates maladaptive drive. Created de novo by the authors using Adobe Illustrator 2025; the figure was not adapted from a previously published illustration.

#### 3.2.3. Network Excitability

Network excitability refers not to the activity of individual neurons alone but to the ability of local neural responses to propagate, synchronize, integrate, and ultimately contribute to system‐level output [64, 65]. In the acute phase, network‐level abnormalities are dominated by SDs, suppression of spontaneous cortical activity, and impaired neurovascular coupling, all of which contribute to lesion expansion [15–17]; concurrently, reduced responsiveness may also occur in distant networks [93]. In the recovery phase, network excitability is increasingly reflected in the restoration of cortical reactivity, reorganization of effective connectivity, and reconfiguration of sensorimotor oscillatory dynamics [64, 66, 94, 95]. Importantly, these changes cannot be reduced to a fixed interhemispheric imbalance model, as their functional meaning depends on lesion location, structural reserve, and residual pathway integrity [96, 97].

#### 3.2.4. Descending Motor‐Drive Excitability

Descending motor‐drive excitability is particularly relevant in patients whose major clinical deficit is impaired motor output. At this level, the critical issue is not whether the cortex itself is more excitable but which descending pathways ultimately gain functional dominance over spinal motor output [97]. In patients with relatively preserved CSTs, recovery is more likely to depend on the reorganization of ipsilesional cortical motor systems [67–70]. By contrast, when corticospinal and corticoreticular pathways are severely compromised, motor output increasingly depends on alternative descending systems, particularly corticoreticulospinal and reticulospinal‐related pathways [74, 75]. These pathways may support residual proximal and gross motor output, but they do not provide an equivalent substitute for corticospinal control of distal individuation and fine motor skill [76, 77]. Therefore, the hyperactive state exhibited by some patients in the chronic phase does not necessarily indicate more refined motor control; if it is accompanied by abnormal coordination, restricted isolated movements, or spasticity, it is more likely to suggest that the system is relying on an output pattern that is easier to recruit but less selective [76, 77]. It is precisely in this sense that, although spasticity and motor recovery both occur within the context of poststroke plasticity, they should not be viewed as variations in the same mechanism; while both are situated within the context of poststroke plasticity, the former is more closely associated with maladaptive descending facilitation and abnormal coordination, whereas the latter is more closely associated with the effective reorganization of the affected‐side corticospinal system [2–17, 43–46, 64–70, 74–78, 93–97].

### 3.3. Three Functional Orientations of Excitability: Injurious, Restorative, and Maladaptive Excitability

Abnormal excitability can arise throughout the course of stroke, but its biological meaning is not constant. Overall, the acute phase is dominated by injurious excitability, in which ionic homeostasis failure, SD, and metabolic collapse promote lesion expansion [4–16, 43–46, 64, 65]. The subacute phase is dominated by restorative excitability, in which surviving circuits become permissive for plasticity, training responsiveness, and functional reorganization [43]. In a subset of patients in the chronic phase, excitability changes further evolve into maladaptive hyperexcitability, characterized by abnormal synergies, spasticity, and low‐selectivity motor output associated with alternative descending drive [2, 77, 78].

### 3.4. The Two Main Axes of This Review: Disease Stage and Anatomical Locus

To avoid conflating excitability changes occurring at different stages, anatomical loci, and mechanistic levels, this review is organized along two intersecting axes: disease stage and anatomical locus. Along the temporal axis, poststroke excitability changes are discussed across the acute, subacute, and chronic phases [4, 5, 98]. Along the spatial axis, we focus on the infarct core and penumbra in the acute phase, the peri‐infarct cortex and bilateral sensorimotor networks in the subacute phase, and the brainstem descending systems and spinal output layer in the chronic phase [2, 78, 96, 99].Within this dual‐axis framework, excitability is further analyzed across four mechanistic levels: membrane, synaptic, network, and descending drive. This approach highlights that poststroke excitability is not a single process but a stage‐dependent and region‐specific redistribution of neural responsiveness (Figure 2).

**Figure 2:**
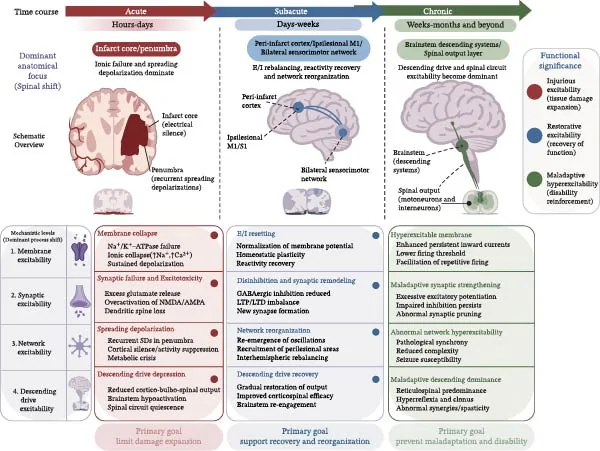
Spatiotemporal shift of the main substrates of poststroke excitability from lesion‐centered injury to network reorganization and maladaptive output. This figure shows the main framework of the review. It combines disease stage, brain region, and mechanistic level. In the acute phase, the main regions are the infarct core and the ischemic penumbra. In these regions, membrane failure, spreading depolarization, and impaired neurovascular coupling cause injurious excitability. In the subacute phase, the main regions shift to the peri‐infarct cortex, the ipsilesional primary motor cortex, and bilateral sensorimotor networks. In these regions, controlled disinhibition, recovery of cortical responsiveness, and network reintegration support restorative excitability. In the chronic phase, in some patients, the main regions further shift to the brainstem‐spinal output system. In this system, alternative descending drive and hyperexcitable spinal output lead to maladaptive hyperexcitability. The bottom axis shows the main mechanistic levels, including membrane, synaptic, network, and descending motor‐drive excitability. Red means injurious excitability. Blue means restorative excitability. Green means maladaptive hyperexcitability. The conceptual distinction between injurious and repair‐associated excitability builds on the framework proposed by Carmichael, whereas the chronic maladaptive component integrates evidence concerning reticulospinal and spinal‐output reorganization [2–4, 78, 99]. Created de novo by the authors using Adobe Illustrator 2025; not adapted from a previously published figure.

## 4. Acute Phase: Injurious Excitability in the Infarct Core and Penumbra

The main problem after acute ischemic stroke is not how surviving networks reorganize but why ischemic tissue quickly changes from relative responsiveness to electrochemical instability and then causes secondary lesion expansion [4]. In this condition, excitability should not be seen simply as an increase in neuronal firing. Instead, it should be understood as an abnormal response of ischemic tissue to ionic imbalance, SD, metabolic mismatch, and impaired neurovascular coupling during severe energy failure [16, 18, 100].

### 4.1. Infarct Core: From Electrical Silence to Sustained Depolarization

In the infarct core, the dominant excitability abnormality is not functional hyperexcitability but the progressive loss of membrane homeostasis caused by severe energy failure. Classic studies of cerebral ischemic thresholds demonstrated that neuronal function and membrane integrity fail at different levels of residual cerebral blood flow (CBF). In human and nonhuman‐primate observations, electroencephalographic and evoked cortical responses become abnormal when CBF falls to ~20 mL/100 g/min and are markedly suppressed or abolished at ~15–17 mL/100 g/min [19, 20]. With more severe ischemia, particularly at flow levels of ~6 mL/100 g/min in classic primate experiments, massive intracellular K^+^ loss and failure of membrane ion homeostasis occur [19]. These values should be interpreted as approximate physiological ranges rather than universal thresholds because they vary according to species, brain region, tissue composition, measurement method, metabolic conditions, and—most importantly—the duration of ischemia [20, 22].

The threshold for irreversible tissue injury is particularly time‐dependent. Experimental observations suggest that CBF values of ~10 mL/100 g/min may lead to irreversible injury when sustained for 1–2 h, whereas the corresponding threshold may be higher during permanent ischemia. Human positron–emission tomography studies performed several hours after stroke onset have reported CBF thresholds for irreversible injury of ~8 mL/100 g/min [22]. Accordingly, a single CBF value cannot independently distinguish reversible dysfunction from irreversible infarction without considering the duration and metabolic context of ischemia.

When oxygen and glucose delivery become insufficient, ATP production decreases, Na^+^/K^+^–ATPase activity fails, and transmembrane ionic gradients progressively collapse [8, 9]. Extracellular K^+^ concentrations rise, while intracellular Na^+^, Cl^−^, Ca^2+^, and water accumulate, driving the membrane potential toward near‐complete depolarization and promoting neuronal and astrocytic swelling [8, 101]. Severe ischemic depolarization is therefore accompanied by suppression of spontaneous and evoked electrical activity rather than by effective neuronal firing [19, 20, 102].

Thus, the apparently “hyperexcitable” condition of the infarct core is more accurately characterized as electrochemical failure: Neurons and glial cells remain persistently depolarized but are unable to generate or transmit physiologically meaningful electrical signals. When repolarization can no longer be restored, this state progresses toward terminal depolarization, ATP depletion, cytotoxic swelling, and irreversible cellular injury [18, 102]. In this context, SD is not merely an associated electrophysiological event but a direct manifestation of gray‐matter membrane failure.

### 4.2. Penumbra: SD and Dynamic Lesion Expansion

Unlike the infarct core, the ischemic penumbra does not immediately lose all metabolic capacity; its defining characteristic is that electrical activity is suppressed, but energy metabolism has not yet ceased entirely [19–21]. Therefore, it is theoretically reversible and serves as the primary target for acute‐phase treatment [22, 118]. However, the penumbra is a dynamic region in a state of unstable equilibrium; its most critical pathological mechanism is not a static state of hypoperfusion but rather recurrent propagating depolarization [18, 23, 24]. SD is a slowly propagating wave characterized by near‐complete depolarization of neurons and glial cells, with a propagation speed of ~2–6 mm/min [18, 24, 25]. In normal brain tissue, SD is typically accompanied by compensatory increases in blood flow; however, under ischemic condition, each depolarization wave increases energy demand, disrupts ionic gradients, aggravates cellular swelling, and further compromises local perfusion, thereby progressively shifting tissue from reversible dysfunction toward irreversible infarction [26–28]. Both clinical and animal studies have shown that the incidence and duration of SD are closely associated with an increase in infarct volume and poor neurological outcomes [29, 30]. The penumbra should therefore be viewed not as a static reservoir awaiting reperfusion but as a region of ongoing pathophysiological recruitment in which excitability abnormalities actively drive lesion expansion [16].

### 4.3. From Ionic Collapse to Cellular Swelling: The Fluid Microenvironment of Acute Excitability Abnormalities

Acute excitability abnormalities are not confined to neuronal membranes and synapses but are deeply embedded in alterations of the brain fluid microenvironment [31, 32, 103]. During ischemia, Na^+^/K^+^–ATPase failure, together with Na^+^, Cl^−^, and water shifts across cellular compartments, causes rapid swelling of neurons and astrocytes [8, 16, 33]. At the same time, extracellular space volume decreases, tortuosity increases, and cerebrospinal fluid/interstitial fluid exchange becomes disturbed, further modifying the diffusion environment for ions, metabolites, and signaling molecules [34]. These changes should not be understood as passive osmotic water uptake alone but as the combined consequence of ionic redistribution, ion–water cotransport, and impaired fluid clearance [35]. Cellular swelling, astrocytic endfoot enlargement, and local pressure changes can then exacerbate microcirculatory dysfunction and further worsen tissue perfusion [36, 37, 103]. In parallel, anaerobic metabolism, lactate accumulation, and tissue acidosis intensify cellular stress and may activate acid‐sensitive channels, such as acid‐sensing ion channel 1a (ASIC1a), thereby increasing Ca^2+^ loading and accelerating neuronal injury [38–40]. Thus, the acute‐phase fluid microenvironment is not a secondary bystander but a core component of injurious excitability. When the cell’s energy supply is insufficient to restore the ion gradient, the cell is unable to repolarize and eventually enters a state of terminal depolarization, indicating that the pathological process has shifted from reversible injury to irreversible necrosis [16].

### 4.4. Reversal of Neurovascular Coupling: From Reactive Blood Flow to Spreading Ischemia

A key factor in acute lesion growth is the change in the relationship between depolarization and blood flow. In normally perfused tissue, SD is usually followed by a hyperemic response that restores metabolic balance [41]. In an ischemic or metabolically weak tissue, the vascular response can reverse. Then, depolarization is followed by vasoconstriction and further drop in perfusion [16–18, 66–70, 74–78, 93–100]. This process, called inverse neurovascular coupling or spreading ischemia, turns a depolarization event from a temporary disturbance into a cause of further tissue recruitment. In this condition, the tissue that is already underperfused does not get extra energy support after depolarization. The tissue becomes more ischemic, harder to repolarize, and more likely to suffer irreversible injury [27, 36, 42]. The acute phase is thus marked by excitability abnormalities in tissue and a self‐amplifying loop between depolarization and perfusion failure.

### 4.5. Summary: Spatial Gradients of Excitability Abnormalities in the Acute Phase

Taken together, acute‐phase excitability abnormalities display a clear spatial gradient. In the infarct core, excitability is dominated by membrane failure, sustained depolarization, and electrical silence. In the penumbra, it is dominated by recurrent SDs and progressive metabolic destabilization of potentially salvageable tissue. In the surrounding vulnerable tissue, excitability abnormalities reflect the propagation of pathological depolarization and the risk of recruitment into the expanding lesion. Acute poststroke excitability should therefore be understood as injurious excitability, whose primary function is not information processing or adaptive reactivity but the amplification and spatial spread of tissue injury (Figure 3).

**Figure 3:**
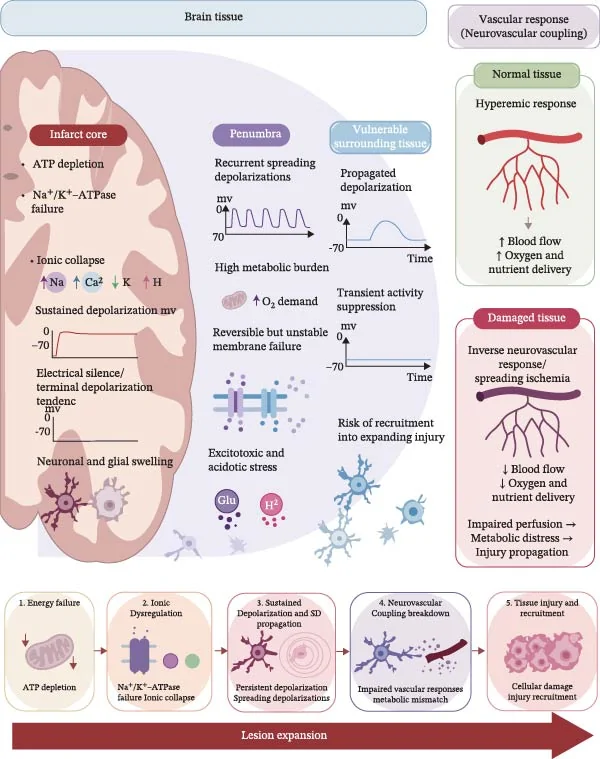
Spatial gradient of injurious excitability in the infarct core and penumbra during acute ischemic stroke. This figure shows the main substrates of acute excitability abnormalities across the ischemic lesion. The infarct core has ATP depletion, Na^+^/K^+^–ATPase failure, ionic gradient collapse, sustained depolarization, electrical silence, and early neuronal and glial swelling. These changes reflect membrane homeostasis failure and not functional hyperexcitability. Around the core, the ischemic penumbra still has some metabolic capacity but is very unstable. Recurrent spreading depolarizations (SDs) cause repeated metabolic stress, worsen ionic imbalance, increase cellular swelling, and gradually recruit salvageable tissue into irreversible infarction. The outer vulnerable tissue zone shows tissue exposed to propagated depolarization and temporary suppression of activity, with risk of further recruitment. The inset on the right compares vascular responses to SD in normal and ischemic tissue. The normally perfused tissue shows compensatory hyperemia. Ischemic tissue can show inverse neurovascular coupling or spreading ischemia, which speeds up lesion expansion. This figure shows that acute poststroke excitability is organized by space and is mainly injurious. Perfusion thresholds and the distinction between electrical failure and persistent depolarization are based principally on Astrup et al. [19] and Hossmann [20]. The propagation and vascular consequences of spreading depolarization are supported by Dreier [24], Hartings et al. [18], Nakamura et al. [29], and Binder et al. [28]. Created de novo by the authors using Adobe Illustrator 2025.

## 5. Subacute Phase: Restorative Excitability in the Peri‐Infarct Cortex and Bilateral Sensorimotor Networks

As the acute metabolic crisis ends, the main question of poststroke neurobiology changes from why tissue is lost to how surviving tissue can be reorganized [4, 5, 47]. The subacute phase is not just a continuation of acute injury. It is not yet a form of chronic stabilization. It is a transitional period where vulnerable but surviving circuits can regain responsiveness and enter a plasticity‐permissive state [4, 43, 46]. In this phase, excitability does not mainly drive lesion expansion. It mainly supports circuit remodeling, training responsiveness, and network reintegration [5, 47, 96].

### 5.1. Peri‐Infarct Cortex: From Hyporesponsiveness to a Plasticity‐Permissive State

The principal substrate for recovery after stroke lies not in necrotic tissue but in the peri‐infarct cortex and its connected regions, where large numbers of neurons remain structurally intact but functionally compromised [4, 5, 98]. Early in the subacute phase, these regions typically exhibit hyporesponsiveness, reduced evoked activity, weakened connectivity, and unstable input–output relationships [4, 50]. This initial low‐reactivity state may partly reflect a protective response that limits metabolic stress in vulnerable tissue. However, if prolonged, it also restricts the ability of surviving circuits to respond to sensory input, behavioral demand, and rehabilitation [4, 43, 96]. Recovery therefore depends not on nonspecific cortical activation but on the gradual transition of peri‐infarct tissue from excessive suppression toward a plasticity‐permissive state in which local responsiveness can again be shaped by experience and training [45, 51–54].

### 5.2. Resetting Synaptic Excitation–Inhibition Balance: From Excessive Tonic Inhibition to Controlled Disinhibition

One of the central mechanisms underlying restorative excitability in the subacute phase is the resetting of local excitation–inhibition balance. In contrast to the acute phase, when synaptic pathology is dominated by glutamate‐mediated excitotoxicity, the subacute phase is increasingly shaped by inhibitory remodeling, particularly excessive extrasynaptic GABA_A receptor–mediated tonic inhibition in peri‐infarct circuits [43, 55]. Compared to phasic inhibition, which is primarily responsible for rapid, time‐dependent postsynaptic inhibition, tonic inhibition manifests as a persistent background brake that reduces a neuron’s input–output gain by setting the action potential threshold and shunting excitatory inputs [56, 57]. This limits the local loop’s ability to respond to experience and training [43]. However, it is important to emphasize that the “disinhibition” referred to in stroke recovery does not simply mean weakening all inhibitory processes but rather a more nuanced process of rebalancing [46]. Current research suggests that excessive tonic inhibition impedes remodeling around the lesion, whereas phasic GABA signaling within specific time windows and at specific levels may be beneficial for recovery [43, 55, 62]. Recovery‐associated disinhibition should therefore not be interpreted as indiscriminate enhancement of excitation. Rather, it reflects a controlled reduction in excessive inhibitory tone that reopens the possibility for selective synaptic strengthening, network reweighting, and experience‐dependent circuit remodeling [43, 46]. In this sense, restorative excitability represents a plasticity‐permissive state rather than a state of generalized hyperactivity.

### 5.3. Recovery of Cortical Reactivity: Changes in Ipsilesional M1 Excitability and Their Limits

During the subacute phase, the most common change in the affected primary motor cortex (M1) is not hyperactivity but rather a gradual recovery of the responsiveness of the motor cortex–CST to stimulation from an initial state of inhibition; this recovery is associated with improved upper limb function, suggesting that the regained recruitability of the affected M1 is a critical step in the transformation of restorative network reorganization into effective motor output [70, 96, 104]. However, the relationship between the two hemispheres cannot be simply summarized as persistent suppression of the affected side by the contralateral side. In a population characterized primarily by subcortical stroke and relative preservation of the affected M1, longitudinal transcranial magnetic stimulation (TMS) studies have not found sustained increases in contralateral M1 excitability or persistently abnormal enhancement of interhemispheric inhibition; on the contrary, recovery is more closely associated with a decrease in the threshold of the affected M1 and the restoration of its responsiveness [96, 105]. By contrast, the role of the contralesional hemisphere is more complex than that predicted by classic interhemispheric imbalance models. Rather than being uniformly detrimental, contralesional activity may be adaptive, neutral, or maladaptive depending on lesion location, structural reserve, and CST integrity: in individuals with good structural reserve, excessive intervention may hinder reorganization on the affected side; whereas in those with severe CST damage, the recruitment of the contralateral secondary motor network may serve a compensatory function [71, 106, 107]. Accordingly, changes in cortical excitability during recovery should not be interpreted through a single interhemispheric rule but through a stratified framework that accounts for residual anatomy and behavioral demands.

### 5.4. Network Reorganization: From Local Disinhibition to Cross‐Regional Reintegration

Restorative excitability in the subacute phase cannot be understood by looking at local circuits alone. If peri‐infarct neurons regain responsiveness, functional recovery is still limited unless these changes are integrated into larger sensorimotor networks. Effective recovery needs two linked processes. Local circuits must respond to input and training. These local changes must also be sent, synchronized, and stabilized across distributed networks [4, 54].

During this period, axonal sprouting, dendritic spine turnover, synaptic remodeling, and changes in interregional connectivity are particularly active, indicating that the nervous system is not merely restoring previous pathways but is actively constructing new routes for functional outputs [58–60]. This also explains why recovery during the subacute phase is highly dependent on experience and training. Restored excitability means that networks are more willing and capable of reallocating weights based on input history. It is important to note that recovery does not equate to a general enhancement of connections across all brain regions; rather, it hinges on the restoration of connections within task‐relevant networks and the reestablishment of normal separation between different networks. At this stage, repetitive use, goal‐directed training, and behavioral shaping are particularly important precisely because they can channel the local plasticity unleashed by disinhibition into functionally meaningful connection strengthening [54, 61]. In the absence of sufficient and task‐specific behavioral input, increased local reactivity does not necessarily lead to high‐quality, task‐relevant network reorganization; rather, it may manifest more as inefficient or compensatory output patterns [61, 108]. This also explains why recovery is highly dependent on experience and task‐specific training. Local reactivity alone is insufficient; it must be embedded in network‐level reintegration to support meaningful behavioral improvement [65, 72, 109].

### 5.5. Dual Constraints of the Subacute Phase: Why the Recovery Window Exists yet Remains Limited

Although the subacute phase represents the most important window for functional recovery after stroke, its value lies precisely in the fact that it is both transient and constrained [4, 110]. First, the peri‐infarct tissue and associated networks, although viable, remain structurally fragile and metabolically vulnerable, meaning that restorative disinhibition must remain tightly regulated [43]. Excessive or poorly timed disinhibition may increase seizure susceptibility or promote inefficient reorganization [63, 111]. Second, the extent of recovery is strongly constrained by the residual structural substrate, including CST integrity, preservation of key motor cortical regions, and reserve within long‐range connections [73]. Third, restorative excitability is strongly time‐dependent [4]. In the weeks to months following stroke, the nervous system enters a highly plastic but temporary period during which interventions are most effective. As this window narrows, networks become increasingly stabilized, spontaneous plasticity declines, and greater training intensity is required to achieve further improvement. Consequently, the requirements for training intensity and duration to achieve functional improvement become significantly more demanding [4, 112].

The subacute phase can be described as follows. The nervous system has not fully recovered from acute injury. It has not yet entered the structural consolidation of the chronic phase. It is in an intermediate stage. This stage is the most responsive to intervention. It also needs precise regulation [92, 113]. If intervention is not enough, local hyporesponsiveness and breakdown of neural networks can become fixed. This can limit later recovery. If intervention is poorly directed or training intensity and timing are not balanced, it may not promote true functional reconstruction. Instead, changes stay mostly in inefficient compensatory output patterns [114–116]. Restorative excitability in the subacute phase is not a static state. It is a dynamic process that must be shaped by behavioral experience, training, and possible neuromodulatory strategies.

### 5.6. Summary: Restorative Significance of Excitability Changes Across Cortical Regions

Overall, subacute‐phase excitability changes are fundamentally different from the injurious excitability that dominates the acute phase. The key question is no longer whether tissue is metabolically collapsing but whether surviving cortical regions and their associated networks can regain sufficient responsiveness to support experience‐dependent reorganization. The peri‐infarct cortex provides the substrate for local plasticity, ipsilesional M1 reestablishes output recruitability, the contralesional hemisphere forms part of a dynamic regulatory background, and distributed sensorimotor networks determine whether local reactivity can be translated into functional recovery. Subacute excitability is therefore best understood as restorative excitability, that is, reactivity reenabled and redirected toward adaptive reorganization (Figure 4).

**Figure 4:**
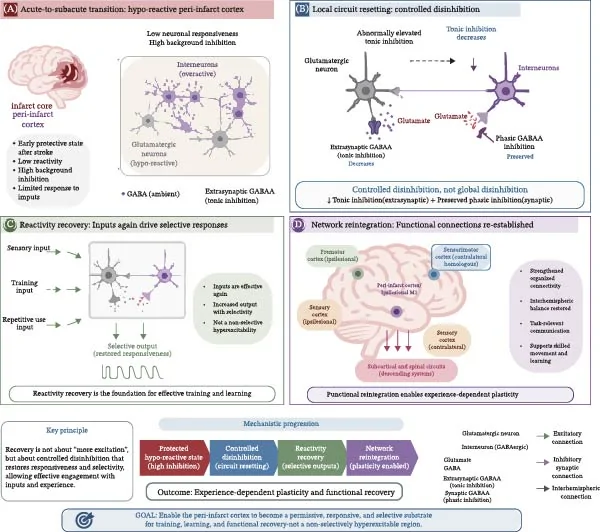
Restorative excitability in the subacute phase: from peri‐infarct disinhibition to ipsilesional reactivity recovery and network reintegration. This figure summarizes the principal mechanisms underlying restorative excitability during the subacute phase after stroke. (A) Shows the early subacute state of the peri‐infarct cortex, in which surviving neurons remain structurally preserved but functionally hyporesponsive, with reduced local reactivity and elevated background inhibition. (B) Shows local circuit resetting. It highlights a controlled reduction of excessive tonic inhibition by extrasynaptic GABA_A receptors. Sufficient inhibitory control is kept for network stability. This change creates a plasticity‐permissive state. It does not cause indiscriminate hyperactivity. (C) Shows the recovery of ipsilesional M1 recruitability. Sensory, behavioral, and training‐related input can again produce effective and more selective motor responses. (D) Shows network reintegration. Local plasticity becomes part of broader bilateral sensorimotor networks through restored functional connections with premotor, somatosensory, and subcortical pathways. The figure emphasizes that subacute excitability is restorative because it supports adaptive circuit remodeling and functional recovery, not merely increased activity. The mechanisms illustrated are based on studies of poststroke tonic and phasic GABAergic inhibition, peri‐infarct plasticity, recovery of ipsilesional cortical reactivity, and sensorimotor network reorganization [43, 46, 50, 64, 96]. Created de novo by the authors using Adobe Illustrator 2025; not adapted from a previously published figure.

## 6. Chronic Phase: Maladaptive Hyperexcitability in Brainstem‐Spinal Output Systems

In the chronic phase, the central problem is no longer whether motor output can be generated but whether it can be generated with sufficient selectivity, fractionation, and stability [2, 96]. Although some patients have regained some degree of active motor function, they gradually develop abnormal coordination, co‐contraction, spasticity, and impaired fine motor skills, suggesting that their residual deficits are not a linear sequela of the acute injury but rather likely reflect maladaptive reorganization against a background of long‐term compensatory output and the dominance of alternative pathways [78, 79, 116]. Current evidence suggests that the onset of spasticity is not entirely synchronized with motor recovery, nor are the two simply different phenotypes of a single mechanism: Motor recovery relies more on the adaptive reorganization of the corticospinal system and related networks [2, 78, 96]. In contrast, spasms and abnormal coordination are more consistent with a framework in which the corticoreticulospinal and reticulospinal pathways dominate and are highly excited [77, 78, 80]. However, these brainstem pathways may also provide some compensation for gross motor function and muscle strength in severely affected patients [81]. Therefore, the key to managing the chronic phase is not simply to suppress all alternative outputs but to reduce their control over abnormal coordination and low‐selective movements, thereby allowing the residual corticospinal control to regain dominance over task‐related movements as much as possible.

### 6.1. Chronic‐Stage Output Reorganization: Insufficient Corticospinal Selectivity and Relative Dominance of Brainstem‐Spinal Drive

The defining feature of chronic abnormal motor output is not persistent generalized cortical hyperexcitability but insufficient reestablishment of highly selective corticospinal control. In some patients, especially those with severe corticospinal and corticoreticular damage, motor output increasingly depends on alternative descending systems, particularly corticoreticulospinal and reticulospinal‐related pathways [96]. A longitudinal study by Stinear et al. [96] conducted during the first 6 months following stroke showed that corticomotor excitability on the affected side was initially suppressed but subsequently recovered and correlated with functional improvement, whereas excitability in the contralateral M1 did not exhibit the sustained increase typically associated with an abnormal chronic motor phenotype; these findings suggest that abnormal motor output in the later stages cannot be simply attributed to increasing cortical excitability. When CST and related corticoreticular control are insufficiently restored, the system may rely more heavily on descending brainstem pathways to maintain gross motor output [81, 82]; however, these pathways are characterized by more diffuse, bilateral, and less selective output [83] and are therefore more likely to be accompanied by abnormal synergy, co‐contraction, flexor bias, and impaired fine motor function [77, 78, 81]. With regard to spasms, the current evidence strongly supports the framework of reticulospinal hyperexcitability and the associated disinhibition of stretch reflexes [2, 84]. However, with regard to the overall phenotype of chronic abnormal movement, a more accurate description would be that it is not that the cortex has completely lost its function but rather that the corticospinal system has failed to reestablish sufficient highly selective control, resulting in the brainstem‐spinal cord drive becoming relatively dominant [78, 96].

### 6.2. How Reticulospinal and Vestibulospinal Imbalance Leads to Stretch Reflex Hyperexcitability

Against this background, one of the most important downstream consequences of chronic excitability reorganization is the persistent elevation of spinal stretch reflex gain [85]. Mechanistically, insufficient restoration of corticospinal and corticoreticular control weakens cortical regulation of inhibitory brainstem systems, thereby allowing a relatively greater influence of facilitatory reticulospinal and vestibulospinal pathways. This further leads to a lowered stretch reflex threshold and increased reflex gain [78, 85, 86]. This shift lowers the stretch reflex threshold, increases reflex gain, and promotes more stereotyped, posture‐linked, and co‐contractive motor output. In this setting, the system is not unable to generate movement; rather, the movement that remains is increasingly shaped by low‐selectivity descending facilitation and hyperexcitable spinal output [78, 83]. Therefore, the issue for patients in the chronic phase is often not a complete loss of motor output but rather that they are still capable of producing motor output, albeit in a manner that is more likely to be postural, co‐contractive, or reflexive.

### 6.3. From Compensatory Output to Maladaptive Hyperexcitability: Why Spasticity Is Not Recovery Itself

Reticulospinal recruitment should not be regarded as pathological in all circumstances, as it may provide useful compensation when corticospinal resources are severely limited [2, 81]. However, when highly selective cortical control fails to recover sufficiently, long‐term reliance on alternative descending drive may evolve into maladaptive hyperexcitability [78]. When corticospinal system resources are insufficient, enhanced reticulospinal tract (RST) activity may help preserve proximal drive, antigravity activities, and even some gross motor output [74, 81, 83]. However, when the cortex fails to maintain precise control over the long term, this alternative output—characterized by reduced selectivity, more diffuse output, greater bilateral involvement, and lower selectivity—increasingly manifests as abnormal coordination, co‐contraction, and impaired segmental movement [83, 87]. McPherson et al. [77] found that the enhancement of synergistic buckling was associated with the progressive recruitment of contralateral corticoreticular spinal pathways, suggesting that the upregulation of alternative pathways is more consistent with motor impairment than with high‐quality recovery. Choudhury et al. [80] further demonstrated that increased output from the RST in patients with chronic stroke is associated with poorer upper limb function, reduced active range of motion in the wrist, and more severe spasticity. Spasticity should therefore not be understood as recovery itself but as a maladaptive consequence of chronic output reorganization in which low‐selectivity descending facilitation becomes relatively dominant [2, 88, 89]. Neurophysiological evidence from the chronic phase further supports this conclusion, it notes that an exaggerated acoustic startle reflex (ASR) is primarily observed in patients in the spastic phase and is not universally present in all patients in the recovery phase; in patients in the flaccid phase and in those who have recovered but do not exhibit spasticity, the ASR typically does not remain persistently elevated [90, 91]. The significance of this phenomenon lies not in simply demonstrating that the brainstem is involved in spasticity but in suggesting that the enhancement of RST is not a universally present, unidirectionally beneficial recovery mechanism in all patients; rather, it is more likely a maladaptive reorganization associated with abnormal coordination, hyperactive stretch reflexes, and a spastic phenotype [2, 89, 99]. In other words, a stronger brainstem system is not necessarily better; when such enhancement is based on damage to or an imbalance in the cortico‐brainstem reticular pathway and insufficient selective control, it is more likely to represent a derailed recovery rather than a successful one.

### 6.4. Summary: The Spatial Focus of Chronic Excitability Abnormalities Shifts From Cortex to Output Systems

In the chronic phase, the dominant substrate of excitability abnormalities is no longer confined to cortical repair zones. Instead, in a subset of patients, it shifts toward the brainstem‐spinal output system, where low‐selectivity descending facilitation and hyperexcitable reflex pathways increasingly shape motor output. The central problem is therefore not persistent cortical overactivity but a shift in the level of control from highly selective corticospinal systems to lower‐resolution output systems. Chronic poststroke hyperexcitability should thus be interpreted less as a sign of successful repair and more as a marker of maladaptive output stabilization, in which movement persists but movement quality deteriorates (Figure 5).

**Figure 5:**
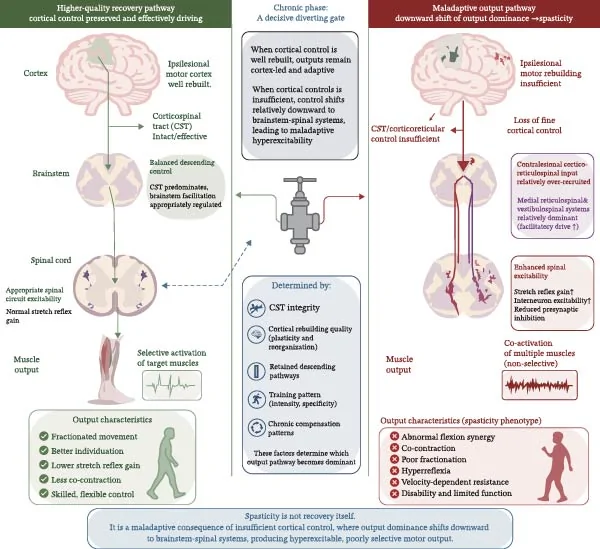
Divergent chronic motor output after stroke: selective corticospinal recovery versus maladaptive brainstem–spinal hyperexcitability. This figure contrasts two distinct chronic‐stage motor outcomes determined by the degree of corticospinal rebuilding and the relative contribution of alternative descending pathways. The left panel depicts a higher‐quality recovery pathway, in which ipsilesional cortical control and corticospinal tract (CST) output are sufficiently preserved or reestablished to support fractionated movement, better individuation, lower stretch reflex gain, and minimal co‐contraction. The right panel depicts a maladaptive pathway, in which insufficient recovery of selective corticospinal and corticoreticular control leads to relative over‐recruitment of corticoreticulospinal, reticulospinal, and related brainstem–spinal output systems. This shift promotes abnormal flexion synergy, co‐contraction, poor fractionation, exaggerated stretch reflex gain, and spasticity. The central gate summarizes the major determinants of chronic motor outcome, including CST integrity, quality of cortical rebuilding, residual descending pathways, training pattern, and long‐term compensation. This divergence explains why preserved movement output does not necessarily indicate restoration of selective motor control. The proposed pathways are based on evidence concerning corticospinal tract integrity, corticoreticulospinal recruitment, reticulospinal output, abnormal motor synergies, and poststroke spasticity [2, 76–78, 80]. Created de novo by the authors using Adobe Illustrator 2025; not adapted from a previously published figure.

## 7. Discussion

### 7.1. Poststroke Excitability Is Not Unidimensional but a Staged, Regional, and Multilevel Redistribution Process

Poststroke excitability abnormalities should no longer be reduced to a simple increase or decrease in neural activity. Instead, they are better understood as a dynamic redistribution of neural responsiveness across different stages, anatomical loci, and mechanistic levels. In the acute phase, excitability abnormalities are dominated by the infarct core and penumbra and primarily promote lesion expansion. In the subacute phase, the dominant substrates shift toward the peri‐infarct cortex and bilateral sensorimotor networks, where excitability becomes increasingly linked to plasticity and functional recovery. In the chronic phase, in a subset of patients, the dominant substrates shift further toward the brainstem–spinal output system, where excitability abnormalities are increasingly associated with maladaptive motor output rather than successful repair [4, 92].

### 7.2. Injury, Recovery, and Maladaptation Are Not Simply Variations in Intensity Along the Same Line

In this view, injurious, restorative, and maladaptive excitability are not just stronger or weaker forms of the same thing. They are different kinds of activity. Injurious excitability is harmful. It speeds up tissue loss through membrane damage, SD, and poor blood flow. Restorative excitability is helpful. It lowers too much inhibition, partly restores cortical responses, and allows networks to reorganize based on experience. Maladaptive hyperexcitability is different. Low‐selectivity descending signals and overly excitable reflexes become stable. This keeps movement but lowers movement quality [2, 4, 43, 46].

### 7.3. The Subacute Phase Is the Critical Window in Which Plasticity Remains Both Accessible and Modifiable

The subacute phase is particularly important. During this period, the surviving tissue still has a strong capacity for reorganization, yet it has not reached the stable state of the chronic phase. Plasticity is therefore both possible and responsive to clinical interventions. This stage is not simply a time of overall increased excitability. Instead, the responsiveness of local circuits and networks can be shaped by behavioral experience, training intensity, and neuromodulatory interventions. The key is not to raise excitability indiscriminately but to guide surviving circuits into a controlled state that permits functional plasticity. If interventions are mistimed, misdirected, or insufficiently targeted, local disinhibition may not lead to meaningful network reorganization and may remain limited to inefficient compensatory activity [54, 111].

### 7.4. Chronic Abnormal Motor Output Reflects a Shift in Control Level Rather Than Persistent Generalized Cortical Hyperexcitability

Chronic motor deficits are not simply due to sustained cortical hyperexcitability. Instead, precise corticospinal control remains incomplete, and movement increasingly depends on alternative descending pathways, particularly corticoreticulospinal and reticulospinal circuits. This shift explains why patients may recover gross movement while still showing abnormal synergies, co‐contraction, hyperactive stretch reflexes, and spasticity. Thus, chronic dysfunction reflects the reliance on low‐selectivity motor pathways rather than the absence of movement. Recognizing this distinction is critical for therapy, highlighting the need to improve the specificity and quality of motor output rather than merely increasing overall activity [77, 78, 80, 83, 96].

### 7.5. Current Limitations and Future Directions

Despite its integrative value, this framework also has important limitations. First, different measures of excitability reflect different biological processes. Membrane instability, synaptic inhibition, cortical reactivity, network connectivity, and reflex gain cannot be treated as equivalent. Second, much of the evidence linking acute excitability changes to lesion expansion comes from animal studies or neurocritical care monitoring, which limits direct application to human patients. Third, direct evidence for chronic reorganization of the brainstem‐spinal output system in humans is still incomplete, and many interpretations rely on indirect electrophysiological or imaging markers. Future studies should move beyond simply describing increases or decreases in excitability. Instead, they should develop longitudinal, multilevel maps that integrate disease stage, anatomical location, circuit organization, and functional outcomes. This approach could make excitability not only a descriptive measure but also a mechanistically guided target for precision interventions [5, 96, 117]. In addition, this study was designed as a structured narrative review rather than a systematic review or meta‐analysis. Although explicit eligibility criteria were applied and the numbers of screened and included studies were reported, selection bias cannot be completely excluded because study selection and evidence synthesis were guided by the conceptual scope of the review. Moreover, human clinical studies, in vivo animal models, and in vitro or ex vivo preparations address different mechanistic levels and should not be interpreted as directly equivalent forms of evidence.

## 8. Conclusion

Changes in excitability after ischemic stroke are not isolated events in a single region. Rather, they reflect a continuous process involving spatial redistribution, mechanistic reorganization, and functional transformation over time. In the acute phase, excitability abnormalities are concentrated in the infarct core and penumbra, where membrane failure, SD, extracellular ionic disturbance, and disrupted neurovascular coupling drive lesion expansion, representing injurious excitability. In the subacute phase, the focus shifts to the peri‐infarct cortex and bilateral sensorimotor networks, where controlled disinhibition, partial recovery of cortical responsiveness, and network reorganization may support restorative excitability. In the chronic phase, in some patients, the brainstem‐spinal output system becomes increasingly prominent. Here, recruitment of alternative descending pathways may contribute to abnormal synergies, spasticity, and low‐selectivity motor control, reflecting maladaptive hyperexcitability.

Poststroke excitability should therefore not be viewed as simply increased or decreased. It must be interpreted in relation to timing, anatomical location, and the mechanistic level involved. From this perspective, excitability becomes more than a descriptive electrophysiological measure; it provides a mechanism‐based framework for understanding stroke progression from injury to recovery or maladaptation. This framework may help guide stage‐specific neuroprotection, plasticity‐oriented rehabilitation, and interventions aimed at reducing maladaptive motor output.

## Author Contributions

Lu Li wrote the manuscript. Le Xie and Dahua Wu designed the framework and revised the manuscript. Hongmei Shi, Lin Huang, Shanshan Zeng, Xiongxing Sun, and Miaomiao Yang jointly conducted literature searches, screening, and data integration.

## Funding

This work was supported by the Key Research Project of the Hunan Provincial Health Commission (Grant 20256456) and the Hunan Provincial Natural Science Foundation General Project (Grant 2024JJ5236).

## Disclosure

This material has not been published in whole or in part elsewhere. The manuscript is not currently being considered for publication in another journal. All authors have been personally and actively involved in substantive work leading to the manuscript and will hold themselves jointly and individually responsible for its content. All authors read and approved the final manuscript.

## Conflicts of Interest

The authors declare no conflicts of interest.

## Supporting Information

Additional supporting information can be found online in the Supporting Information section.

## Supporting information


**Supporting Information** Figure S1: Literature identification and study‐selection process. Records were identified through database searches and supplementary reference and citation screening. After duplicate removal, titles and abstracts were screened for relevance, followed by full‐text eligibility assessment. Eligible publications addressing membrane excitability, synaptic excitatory–inhibitory rebalancing, cortical and network reorganization, or descending motor and spinal excitability after ischemic stroke were included in the narrative synthesis. The flow diagram was adapted from the PRISMA 2020 framework. The diagram is presented in a PRISMA‐style format to improve the transparency of literature identification and selection; the review was conducted as a narrative rather than a systematic review.

## Data Availability

Data sharing is not applicable to this article as no datasets were generated or analyzed during the current study.
