# Supplementary material for: Spatiotemporal Remodeling of Neural Excitability Following Ischemic Stroke: Regional Mechanisms From the Infarct Core to Brainstem–Spinal Output Systems: A Structured Narrative Review
Source: Neural Plast. 2026 Oct 29;2026:3095088. doi: 10.1155/np/3095088 (PMC13720517; doi:10.1155/np/3095088)
Supplement: Supplementary file 1 [file NP-2026-3095088-s001.docx]

Figure supplementary 1 Literature identification and study-selection process.

Figure S1. Literature identification and study-selection process. Records were identified through database searches and supplementary reference and citation screening. After duplicate removal, titles and abstracts were screened for relevance, followed by full-text eligibility assessment. Eligible publications addressing membrane excitability, synaptic excitatory–inhibitory rebalancing, cortical and network reorganization, or descending motor and spinal excitability after ischemic stroke were included in the narrative synthesis. The flow diagram was adapted from the PRISMA 2020 framework. The diagram is presented in a PRISMA-style format to improve the transparency of literature identification and selection; the review was conducted as a narrative rather than a systematic review.
